# Comparative Evaluation of Hearing and Salivary Flow Rate in Smokers and Non-smokers: A Cross-Sectional Study

**DOI:** 10.7759/cureus.67794

**Published:** 2024-08-26

**Authors:** Khushi Meghani, Shaila Sidam, Ashish Pakhre, Ananyan Sampath, Anjan K Sahoo, Aparna G Chavan

**Affiliations:** 1 Otolaryngology - Head and Neck Surgery, All India Institute of Medical Sciences, Bhopal, Bhopal, IND; 2 Psychiatry, All India Institute of Medical Sciences, Bhopal, Bhopal, IND; 3 Medical College, All India Institute of Medical Sciences, Bhopal, Bhopal, IND

**Keywords:** salivary flow rate, saliva, hearing, smoking, audiometry

## Abstract

Background

Smoking is a major global health issue that is linked to various health conditions, including hearing loss and reduced salivary flow. This study aims to explore the relationship between smoking, hearing loss, and salivary flow rate.

Methods

This cross-sectional study was conducted over two months at a tertiary healthcare institute in Central India, involving 100 participants (50 smokers and 50 non-smokers) aged 18-55 years. Hearing status was assessed using audiometry, and the salivary flow rate was measured. Statistical analyses were performed using SPSS version 21 (IBM Corp., Armonk, NY, US).

Results

Smokers had a significantly higher prevalence of hearing loss (40%) compared to non-smokers (10%). The salivary flow rate was significantly lower in smokers (mean 0.5540 ml/min) than in non-smokers (mean 0.9240 ml/min). However, no significant correlation was found between the duration and frequency of smoking with hearing loss or salivary flow rate.

Conclusion

Smoking significantly impacts both hearing and salivary flow rate, hence smokers show a higher risk of hearing loss and reduced salivary flow rate. Early hearing screenings and preventive measures are recommended for smokers. Further research with larger sample sizes is needed to explore the long-term effects of smoking on these health parameters.

## Introduction

Cigarette smoking is a widespread habit globally, with approximately 1.3 billion smokers [1]. The detrimental effects of tobacco on health are not always immediately obvious, often taking years to become apparent. Despite being the leading cause of death, it can be reduced by taking timely measures to reduce this epidemic.

Smoking has negative effects on overall health and well-being, including decreased physical fitness, increased risk of infection, and impaired wound healing [2]. Besides these, smoking is one of the risk factors for hearing loss and developing oral conditions like tooth discoloration, dryness, oral lesions, candida infections, halitosis, and having a seductive and dependent effect [3,4]. It is estimated that smokers have 1.69 times more risk of hearing impairment than non-smokers [5]. Smoking causes oxidative stress, which can lead to changes in blood vessels and melanin levels, further increasing the risk of hearing impairment [6,7].

Saliva is a complex body fluid essential for oral health [8]. The reduced salivary flow rate can lead to oral and dental issues. Overall hearing, dental, and oral problems can reduce the quality of life by affecting physical and social performance along with depressive symptoms and reduced self-confidence [4].

Studies have shown a positive association between smoking and hearing loss [9]. Evidence suggests smoking to be one of the external factors that reduce the salivary flow rate; however, research findings are contrasting [10,11]. There are conflicting reports about the correlation between cigarette smoking and reduced salivary flow rate, as some studies have indicated that cigarette smokers have lower salivary flow rate than non-smokers [3,4,12], whereas other studies have shown that cigarette smoking does not affect the salivary flow rate [11,13]. Hence, considering the controversies and knowledge gap due to limited studies regarding the association of smoking with salivary flow rate and hearing status, this study has been conducted.

## Materials and methods

This prospective cross-sectional study was conducted in the Outpatient Department (OPD) of ENT & Head and Neck Surgery of a tertiary healthcare institute in Central India over a period of two months. Ethical clearance was obtained from the Institutional Ethical Committee vide reference number AIIMS/BPL/IECSR/JAN/23/STS/03 prior to participant selection.

Due to logistic reasons and the predetermined time allotted for completion of the study (six months), a feasibility-based approach was used to collect data from a total of 100 participants (50 per group). Participants aged between 18 and 55 years were selected from the OPD, as this is the common age range visiting the OPD with a similar clinical presentation to involve a larger pool of eligible patients. They were later classified as smokers (i.e. past or current smokers) and non-smokers (i.e. who have never smoked).

The inclusion criteria for the study were that both the study and control groups comprised individuals over 18 years of age who consented to participate, with the study group specifically including individuals who smoked at least two cigarettes per day for more than six months. The exclusion criteria ruled out individuals with systemic illnesses that could alter salivary flow rates, such as diabetes mellitus, salivary gland dysfunction, immunocompromised conditions, autoimmune diseases, and Sjögren’s syndrome. Additionally, smokers with a history of using ototoxic drugs, any form of hearing loss, severe or recurrent ear infections, ear surgery, hypertension, occupational exposure to loud noises daily, history of oral reparative procedures, or ulcerative lesions in the oral cavity were excluded from the study.

Participants in both the study and control groups were selected randomly according to predefined inclusion and exclusion criteria. All individuals willingly took part in the study and informed, written consent was obtained from each one.

**Figure 1:**
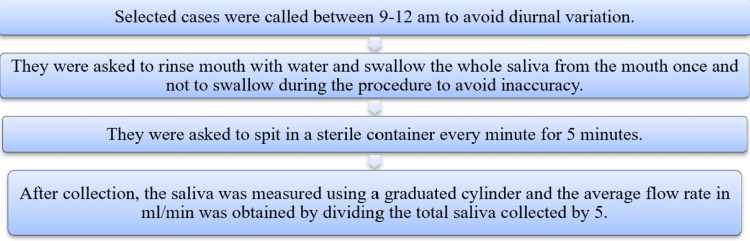
Procedure followed for the test

A detailed case history was taken for demographic details, medical history, addiction, and drug history. Details regarding the duration and frequency of smoking were also noted, followed by an oral examination. The participants were also informed about the conduction of the procedure (Figure 1).

Audiometry

Hearing tests were done in a soundproof room in the ENT OPD. The hearing examination included otoscopy, a screening tuning fork test, and pure tone audiometry. The tuning fork test was done using a 512 Hz tuning fork. Audiometry was done using the Maico MA 42 Puretone portable audiometer (MAICO Diagnostics, Eden Prairie, MN, US).

Statistical analysis

Statistical analysis was done using SPSS version 21 (IBM Corp., Armonk, NY, US). Mean and standard deviation were calculated and a comparison of flow rate between smokers and non-smokers was done using an unpaired t-test. The analysis of variance (ANOVA) test was used to compare the duration and frequency of smoking with the flow rate among smokers. A p-value <0.05 was considered significant.

## Results

**Figure 2:**
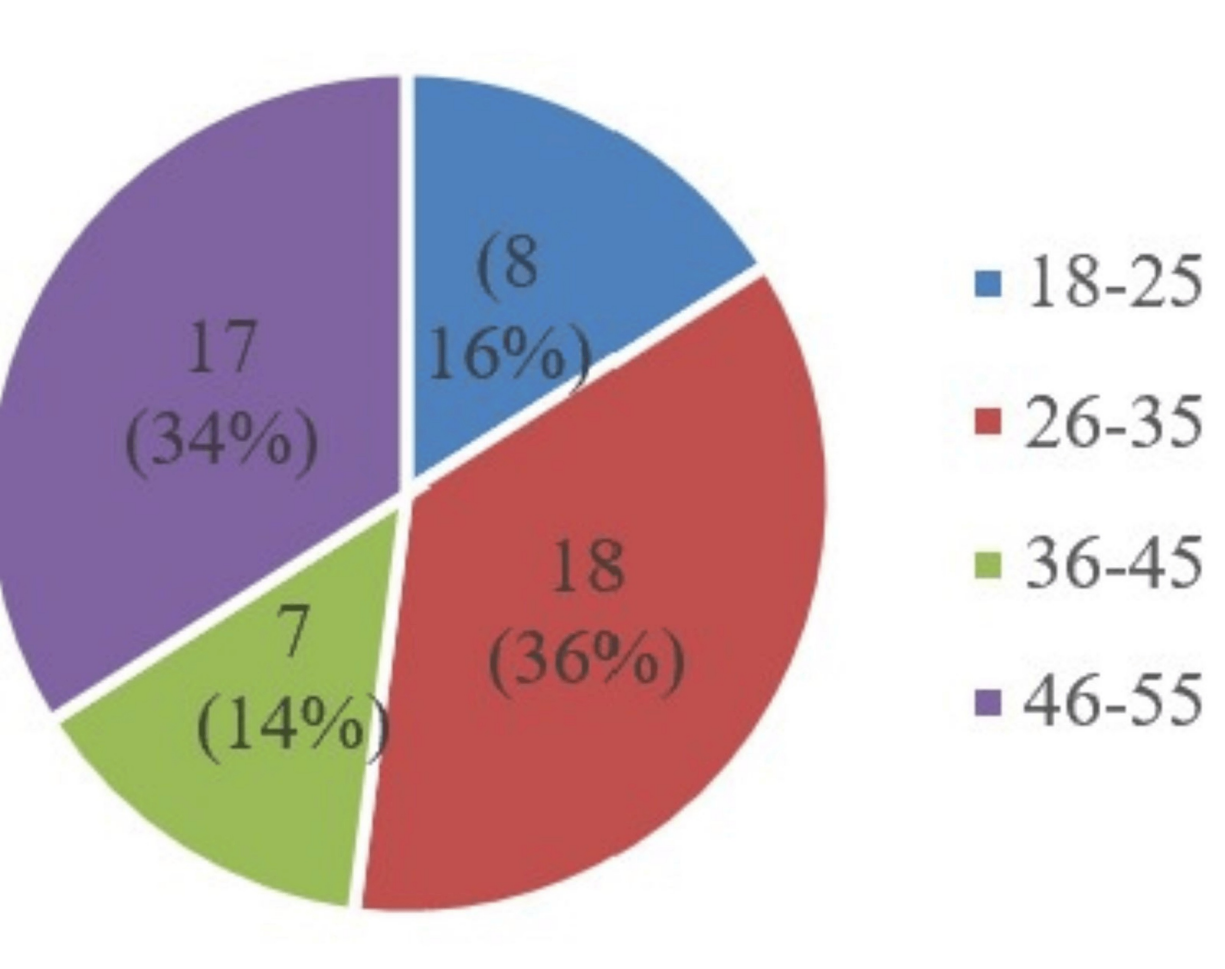
Age distribution of smokers

Figure 2 depicts the number of smokers in various age groups, with the highest in the age group of 26-35 (18; 36%) followed by the 46-55 age group (17; 34%), the 18-25 age group (8; 16%), and the 36-45 age group (7; 14%).

**Figure 3:**
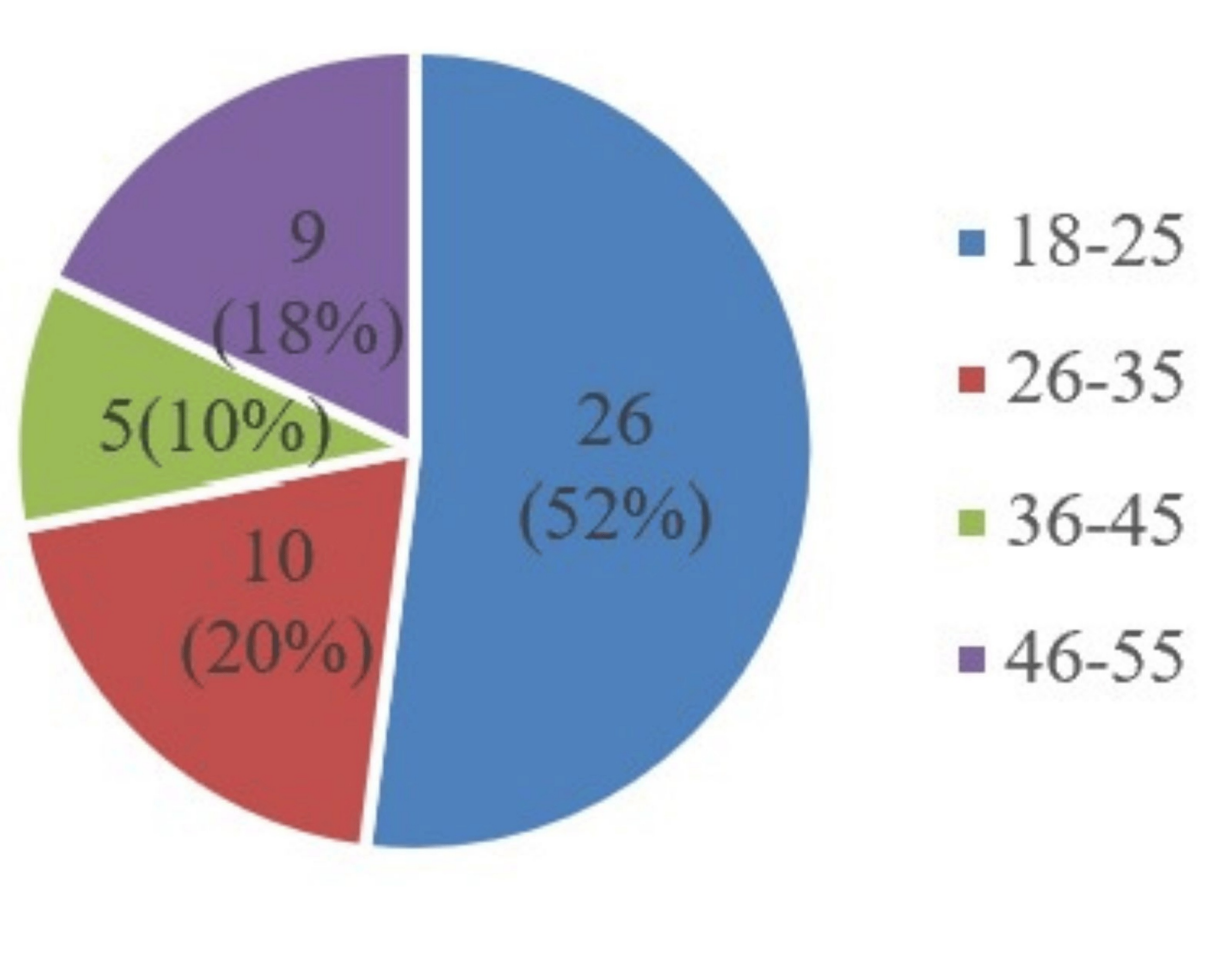
Age distribution of non-smokers

Figure 3 depicts the number of non-smokers in various age groups, with the highest in the age group of 18-25 (26; 52%), followed by the 26-35 age group (10; 20%), the 46-55 age group (9; 18%), and the 36-45 age group (5; 10%).

The difference between the mean age of smokers (37.84 ± 12.038) and non-smokers (30.30 ± 11.560) was statistically significant. Out of 50 non-smokers, 14 (28%) non-smokers were female and 36 (72%) were male. All the smokers were male.

As per Table 1, 30 (60%) out of 50 smokers were found to have no hearing loss while 45 (90%)out of 50 non-smokers were unaffected. Smoking was found to be statistically associated with hearing loss with 20 (40%) smokers and 5 (10%) non-smokers having hearing impairment (p-value 0.004). More smokers were found to have mild hearing loss, i.e. 9 (18%) smokers, and fewer with severe hearing loss, i.e. 5 (10%) smokers. Table 1 also depicts the degree of hearing loss in different age groups. Out of a total of 20 affected smokers, 11 (55%) were from the oldest age group (46-55 years) with 7 (35%) individuals having a mild degree of hearing loss and the remaining (4; 20%) having a moderate degree of hearing loss.

**Table 1:** Degree of hearing loss in smokers and non-smokers of different age groups using the chi-square test (p-value <0.05 considered significant)

Smoking status	Age (in years)	No hearing loss	Affected subjects	Total	Degree of hearing loss			p-value*
					Mild (26-40 dB)	Moderate (41-60 dB)	Severe (>60 dB)	
Smokers	18-25	6 (12%)	2 (4%)	8 (16%)	0	1	1	0.004
	26-35	13 (26%)	5 (10%)	18 (36%)	2	0	3	
	36-45	5 (10%)	2 (4%)	7 (14%)	0	1	1	
	46-55	6 (12%)	11 (22%)	17 (34%)	7	4	0	
	Total	30 (60%)	20 (40%)	50 (100%)	9	6	5	
Non-smokers	18-25	25 (50%)	1 (2%)	26 (52%)	1	0	0	
	26-35	10 (20%)	0	10 (20%)	0	0	0	
	36-45	5 (10%)	0	5 (10%)	0	0	0	
	46-55	5 (10%)	4 (8%)	9 (18%)	3	1	0	
	Total	45(90%)	5 (10%)	50(100%)	4	1	0	

Smoking status	Age (in years)	No hearing loss	Affected subjects	Total	Degree of hearing loss			p-value*
					Mild (26-40 dB)	Moderate (41-60 dB)	Severe (>60 dB)	
Smokers	18-25	6 (12%)	2 (4%)	8 (16%)	0	1	1	0.004
	26-35	13 (26%)	5 (10%)	18 (36%)	2	0	3	
	36-45	5 (10%)	2 (4%)	7 (14%)	0	1	1	
	46-55	6 (12%)	11 (22%)	17 (34%)	7	4	0	
	Total	30 (60%)	20 (40%)	50 (100%)	9	6	5	
Non-smokers	18-25	25 (50%)	1 (2%)	26 (52%)	1	0	0	
	26-35	10 (20%)	0	10 (20%)	0	0	0	
	36-45	5 (10%)	0	5 (10%)	0	0	0	
	46-55	5 (10%)	4 (8%)	9 (18%)	3	1	0	
	Total	45(90%)	5 (10%)	50(100%)	4	1	0	

Table 2 shows the relation of hearing loss with the number of bidis/cigarettes smoked per day. The association between severity and number of bidis/cigarettes smoked per day was not found to be statistically significant (p-value 0.132). As per Table 2, people who smoked for a longer duration were affected more as compared to those who smoked for a shorter duration. However, this relation between the severity of hearing loss and the duration of smoking in years was not found to be statistically significant (p-value 0.295).

**Table 2:** Relation of hearing loss with the number of bidis/cigarettes per day or with the duration of smoking using the chi-square test (p-value <0.05 considered significant)

Number of Bidi / Cigarettes per day	No hearing loss	Affected subject	Degree of hearing loss			Total	p-value*
			Mild (26-40 dB)	Moderate (41-60 dB)	Severe (>60 dB)		
1-10	16 (32%)	14 (28%)	5 (10%)	5 (10%)	4 (8%)	30 (60%)	0.132
11-20	13 (26%)	3 (6%)	1 (2%)	1 (2%)	1 (2%)	16 (32%)	
21-30	1 (2%)	2 (4%)	2 (4%)	0	0	3 (6%)	
>30	0	1 (2%)	1 (2%)	0	0	1 (2%)	
Total	30 (60%)	20 (40%)	9 (18%)	6 (12%)	5 (10%)	50 (100%)	
Duration of smoking (in years)							
< 5	10 (20%)	4 (8%)	1 (2%)	1 (2%)	2 (4%)	14 (28%)	0.295
5-10	11 (22%)	4 (8%)	1 (2%)	1 (2%)	2 (4%)	15 (30%)	
11-20	4 (8%)	8 (16%)	4 (8%)	3 (6%)	1 (2%)	12 (24%)	
21-30	5 (10%)	3 (6%)	2 (4%)	1 (2%)	0	8 (16%)	
>30	0	1 (2%)	1 (2%)	0	0	1 (2%)	
Total	30 (60%)	20 (40%)	9 (18%)	6 (12%)	5 (10%)	50 (100%)	

On comparing the salivary flow rate among smokers and non-smokers, the mean salivary flow rate in smokers (0.5540 ± 0.2908 ml/min) was less as compared to non-smokers (0.9240 ± 0.3153 ml/min). This difference observed was statistically highly significant with a p-value of <0.001 (Table 3).

**Table 3:** Comparison of salivary flow rate in smokers and non-smokers using an unpaired t-test (p-value* < 0.05 considered significant)

Smoking status	N	Salivary flow rate (ml/min)				
		Mean	Std. Deviation	t	DF	p-value*
Smokers	50 (100%)	0.5540	0.29082	-6.099	98	<0.001
Non-smokers	50 (100%)	0.9240	0.31530			

On comparison of the frequency of smoking with salivary flow rate, it was found that the mean SFR in smokers who smoked 21-30 cigarettes/bidis per day was 0.2667 ± 0.11540 ml/min in comparison to smokers who smoked less than 10 cigarettes per day where the mean SFR was 0.5400 ± 0.24719 ml/min. This difference was found to be statistically significant with a p-value of 0.036 (Table 4). The duration of the smoking habit was compared with the salivary flow rate. It was observed that the mean salivary flow rate in smokers smoking less than 5 years (0.6143 ± 0.2537 ml/min) as compared to the mean salivary flow rate in smokers with a smoking history of 5-10 years (0.4600 ± 0.18349 ml/min). This relation of the duration of smoking habit with salivary flow rate was not statistically significant (p-value 0.345) (Table 4).

**Table 4:** Comparison of salivary flow rate with the number of bidis/cigarettes smoked per day and with the duration of smoking using the one-way ANOVA test (p-value* < 0.05 considered significant) ANOVA: analysis of variance

Number of bidi/ cigarettes per day	N	Salivary flow rate (ml/min)				
		Mean	Std. Deviation	F	DF	p-value*
1-10	30 (60%)	0.5400	0.24719	3.096	49	0.036
11-20	16 (32%)	0.5938	0.33160			
21-30	3 (6%)	0.2667	0.11547			
>30	1 (2%)	1.2				
Total	50 (100%)	0.5540	0.29082			
Duration of smoking (in years)						
<5	14 (28%)	0.6143	0.25376	1.150	49	0.345
5-10	15 (30%)	0.4600	0.18439			
11-20	12 (24%)	0.5667	0.26742			
21-30	8 (16%)	0.6500	0.48697			
>30	1 (2%)	0.2000				
Total	50 (100%)	0.5540	0.29082			

## Discussion

This prospective cross-sectional study examined 50 smokers and 50 non-smokers aged 18-55 years to assess the impact of smoking on hearing and salivary flow rate. It was found that smoking significantly contributes to hearing loss, with smokers showing a higher prevalence of hearing impairment compared to non-smokers (p-value 0.004). This finding aligns with previous studies done by Cruickshanks KJ et al., Kumar A et al., and Gautam N et al. [14-16]. The study also highlighted that age and smoking have a multiplicative effect on hearing loss, with an increasing percentage of affected individuals in both groups as age progresses, which was in line with other studies such as Noorhassim I et al., Cruickshanks KJ et al., and Kumar A et al. [14,15,17].

Contrary to some studies, this research did not find a statistically significant relationship between the duration or frequency of smoking and hearing loss. This discrepancy may be attributed to the small sample size. Other studies have suggested that long-term and heavy smoking exacerbates the risk of hearing loss, emphasizing the need for further research with larger sample sizes to confirm these findings [9,15,18].

In terms of salivary flow rate, the study found a significant reduction in smokers compared to non-smokers. The mean salivary flow rate was lower in smokers, and the reduction correlated with the number of cigarettes smoked per day (p-value 0.036). However, no significant relationship was found between the duration of smoking and salivary flow rate, again potentially due to the small sample size.

The pathophysiological mechanisms by which smoking affects hearing and salivary flow include the vasoconstrictive effects of nicotine, which reduce blood flow to the cochlea and salivary glands. Additionally, the oxidative stress and inflammation caused by smoking can damage the auditory and salivary tissues. The presence of ototoxic chemicals in tobacco smoke further exacerbates the risk of hearing loss.

The limitation of the study is that it had a small sample size, which may be the reason for some statistically insignificant results. Considering the age range of the sample and its impact on health issues, the findings should be considered under a potential age-related bias. We did not get female patients for the study sample, and this may also impact the findings and their implications. Also, this study has not considered secondhand smoke. Future research should address these limitations by including larger and more diverse sample sizes and considering the effects of passive smoking.

## Conclusions

The findings of this study demonstrate that smoking significantly impacts hearing and salivary flow rate. Smokers are more likely to experience hearing loss and reduced salivary flow compared to non-smokers. While the study did not find a significant relationship between the duration and frequency of smoking with hearing loss, it did establish that the frequency of smoking adversely affects the salivary flow rate.
